# Parent-adolescent discussion about reproductive health issues and associated factors among parents in Debre Markos town, Northwest, Ethiopia: a cross-sectional study

**DOI:** 10.11604/pamj.2022.41.266.32447

**Published:** 2022-04-01

**Authors:** Sefanit Sleshi, Addisu Alehegn Alemu, Zewdu Dagnew, Bewket Yeserah Aynalem

**Affiliations:** 1 Health Science College, Debre Markos University, Debre Markos, Ethiopia

**Keywords:** Adolescents, Debre Markos, discussion, parents, reproductive health

## Abstract

**Introduction:**

adolescence is a transition phase from being a child to an adult. Open positive parent-adolescent communication on reproductive health issues has many positive effects on adolescents, families, and society.

**Methods:**

a community-based cross-sectional study design was employed, and a multistage sampling technique was used. Data were collected through face-to-face interviews with pre-tested structured questionnaires. After data collection, data were coded and entered using Epi data version 3.1 and analysed using SPSS version 25 statistical software. Binary logistic regression analysis was used to ascertain the association between explanatory variables and the outcome variable. Variables with a P value less than 0.25 in the bivariable analysis and P-value < 0.05 in the multivariable analysis and corresponding 95% CI of odds ratio were considered to declare a result as statistically significant.

**Results:**

this study has revealed parent-adolescent discussion on reproductive health issues was 55.2%. Age 45-54 (AOR=2.37, 95% CI: 1.28-4.39) and 55-64 (AOR=2.54, 95% CI: 1.15-5.56) years, male parents (AOR= 0.51, 95% CI: 0.29-0.89) and monthly income above 158 USD (AOR=3.31, 95% CI: 1.79-6.12) were statistically significant.

**Conclusion:**

more than half of the parents discuss reproductive health issues with adolescents. Age 45-54 and 55-64 years, male parents, and higher incomes were the factors that allowed parent-adolescent discussion on reproductive health issues.

## Introduction

Adolescence is a period in which an individual undergoes major physical and psychological changes and it is a phase in which an individual is no longer a child but is not yet an adult, which is a transition from being a child to an adult [1, 2]. Adolescents are susceptible to sexual risk-taking because of social pressures, mixed messages about sexuality and Reproductive Health (RH), as well as having limited resources, or support to protect themselves from unsafe sex [3, 4], and the use of tobacco, alcohol, or illicit drugs, even casually, increases the chance that an adolescent will engage in high-risk sexual behaviours [5, 6]. Open positive parent-adolescent discussion on reproductive health issues has many positive effects on adolescents, families, and society [7, 8]. Furthermore, with the constant efforts by the parents in knowing their teens' friends have fewer sexual partners, fewer coital acts, and more use of condoms and contraceptives among adolescents and they are at a reduced risk from unintended pregnancies, abortion, HIV, and other STIs [9, 10].

The consequences of adolescent sexual risk-taking behaviours are associated with significant costs for both the individual and society as a whole and they result in a new diagnosis for STIs and HIV/AIDS [11, 12]. Teen pregnancy and birth are other major health problems that lead adolescents to great complications [6, 13]. According to the Ethiopian Demographic Health Survey (EDHS), the percentages of adolescent girls who have begun childbearing were 12.4% [14]. There were 23 million adolescents from different parts of Ethiopia engaged in “A Rights-Based Approach to Adolescent and Youth Development, Ethiopia” training, which contained different programs including strengthened capacity of implementing partners for coordination, implementation, monitoring, and evaluation of RH, HIV, gender-responsive programs and improved capacity of parents and communities to respond to demands of young people and ensure a protective and enabling environment, yet even after the utilization of the training results a parent-adolescent discussion on RH issues was low in different parts of Ethiopia [15-21]. There was little or no community-based research made in Amhara Regional State, especially in the East Gojjam zone administration, regarding the parent-adolescent discussion about RH issues. Therefore, this study was conducted to assess the magnitude of parent-adolescent discussion about reproductive health issues and associated factors among parents in Debre Markos town, East Gojjam Zone, Ethiopia.

## Methods

**Study area and period:** the study was conducted from 15 to 30 March 2019 in Debre Markos town which is the capital city of East Gojjam Administrative Zone, Amhara Regional State, Ethiopia. According to a report released by the Debre Markos city administration office in July 2018, the town was inhabited by 125,634 people of whom 58,513 were men, and the number of adolescents aged 10-19 was 30,060 of whom 16,969 were females [22].

**Study Design:** a community-based cross-sectional study design was employed.

### Populations

**Source population:** all parents who were living in Debre Markos town and who had adolescent children during the data collection period.

**Study population:** all selected parents who had adolescent children from the selected Kebeles in Debre Markos town during the data collection period.

**Sample size determination and procedure:** the sample size was calculated by using the single population proportion formula by assuming 28.76% of parents discussed RH issues with their adolescents from the community-based study conducted among parents in Harar, eastern Ethiopia in 2014 [21] with a 95% confidence level and estimated margin of error 5%.


$$n=z\frac{\alpha^{2}}{2}\frac{p(1-p)}{d^{2}}$$

n=(1.96)^2^(0.29)(0.71)/(0.05)^2^=316.3^~^316

Since the multistage sampling method was applied, the design effect of 1.5 was considered. Therefore, the final sample size, with the design effect of 1.5 and the non-response rate of 5%, was 498. There were seven Kebeles (the lowest administrative unit) in Debre Markos town. To assure representativeness in the Kebele level, three Kebeles (Kebele 2, 4, and 7), were selected by using simple random sampling. Then the survey was done to determine the number of households where parents who had adolescent children live in each Kebele. After the survey, the sample size was proportionally allocated to each Kebele based on several households where parents having adolescent children were living in the selected Kebeles. Finally, a systematic random sampling method was used for selecting parents fulfilling the inclusion criteria.

### Variables

**Outcome variable:** parent-adolescent discussion about reproductive health issues

**Independent variables:** socio-demographic characteristics; knowledge of reproductive health; discussion on reproductive health and attitude towards reproductive health discussion.

### Operational definitions

**Discussion on RH issues:** parents who have ever discussed at least two RH issues (contraception, STIs/HIV/AIDS, sexual intercourse, unwanted pregnancy, avoiding premarital sex, condom, changes during puberty, and menstrual cycle) with adolescents living in the household were considered to have discussed on RH issues [17, 19].

**Knowledgeable:** parents who scored points more than the mean score out of prepared knowledge part questions [17].

**Parents:** those who had adolescent children like biological parents, foster parents, step-parents but not including elder siblings [17, 19].

**Positive attitude towards RH discussion:** those respondents who had a positive stance towards RH discussion and who scored points more than the mean score out of prepared attitude questions [17].

**Data collection procedures:** data were collected through face-to-face interviews using a pre-tested questionnaire which was adapted from different kinds of literature. Five unemployed young people in Debre Markos who had completed grade 12 collected the data under the supervision of one B.Sc. Degree holder nurse. The supervisor and the principal investigator made frequent checks on the data collection process to ensure the completeness and consistency of the gathered information and errors found during the process. A written information sheet with a section on informed consent was attached to the questionnaire to ensure all participants get the same directions and information.

**Data quality assurance:** to maintain the quality of the data, a pretest was taken before actual data collection among parents of adolescents in a Kebele which was not selected in Debre Markos. The questionnaire was translated from English into Amharic and back to English by language experts to check for consistency. Data collectors and the supervisor were given training for two days by the principal investigator on the objectives, relevance of the study, ethical consideration, and the techniques of the interview.

**Data analysis and processing:** after data collection, filled questionnaires were coded and entered using Epi data version 3.1 then exported to SPSS version 25 for analysis. Data cleaning was performed to check for accuracy, consistencies, and missed values and variables. Descriptive analysis such as proportions, percentages, means, and measures of dispersion was computed to describe the respondents in terms of socio-demographic characteristics and prevalence of parent-adolescent discussion of RH issues. Binary logistic regression analysis was used to ascertain the association between individual explanatory variables with the outcome variable. Variables that had p values < 0.25 were entered into multivariable analysis to identify associated factors of parent-adolescent discussion on reproductive health issues. Variables having a P-value <0.05 with a corresponding 95% CI were considered statistically significant. The direction and strengths of the association were interpreted from the adjusted odds ratio. Finally, the results were presented in texts and tables.

**Ethical clearance:** ethical approval was first obtained from Debre Markos University research ethical approval committee with ethical approval number HSC/997/16/11. Similarly, official ethical letters were obtained from each Kebeles administration included in the study. Following an explanation of the purpose of the study, consent was obtained from each participant. Confidentiality of information and privacy of participants were assured for all the information provided, to preserve confidentiality the data were not exposed to the third party.

## Results

**Socio-demographic characteristics of parents:** a total of 478 parents had participated in the study, making the response rate 96%. The mean age of the respondents was 41.64±9.63SD with the age range of 26 to 73 years. Nearly three-fourths (74.7%) of the participants were females. Most of the respondents (90.4%) were Orthodox Christianity followers. More than half (56.9%) of the participants have completed primary education and above. Around two-thirds of the participants (65.9%) were married and living together with their spouses (Table 1).

**Table 1:** socio-demographic characteristics of respondents in Debre Markos East Gojjam Ethiopia, March 2019

Variable	Characteristics	Frequency	Percentage
Sex	Female	357	74.7%
	Male	121	25.3%
Religion	Orthodox christian	432	90.4%
	Protestant	31	6.5%
	Muslim	13	2.7%
	Others*	2	0.4%
Educational status	Uneducated	125	26.2%
	Read and write only	81	16.9%
	Primary school	76	15.9%
	Secondary school	69	14.4%
	Diploma	71	14.9%
	Degree and above	56	11.7%
Occupation	Housewife	218	45.6%
	Employed	111	23.2%
	Merchant	109	22.8%
	Others**	40	8.4%
Marital status	Living together	315	65.9%
	Live in separate places	59	12.3%
	Divorced	46	9.6%
	Widowed	58	12.1%
Age	25-34	105	22.0%
	35-45	212	44.4%
	45-54	101	21.1%
	55-64	49	10.3%
	>65	11	2.3%
Income	Less than 53.4 USD	118	24.7%
	53.4-106.8 USD	165	34.5%
	106.8-158 USD	79	16.5%
	Above 158 USD	116	24.3%

* Jehovah Witness, Catholic **Labor worker, Student, Unemployed

**Knowledge, Discussion and attitude towards Reproductive Health Discussion:** about 216 (45.2%) of the participants were knowledgeable (Table 2) and79.3% of the total respondents had a positive attitude (Table 3). Nearly nine out of ten (87.9%) of the participants agreed on the necessity of RH discussion with adolescents, however, only (55.2%) of them had ever discussed at least three components of RH with their adolescent children (Table 4).

**Table 2:** distribution of knowledge of respondents on RH in Debre Markos, East Gojjam Ethiopia, March 2019

Questions Asked	Categories		Frequency	Percentage
Know about STIs	Gonorrhea	Yes	231	79.4%
		No	60	20.6%
	Syphilis	Yes	253	86.9%
		No	38	13.1%
	HIV/AIDS	Yes	226	78.2%
		No	63	21.8%
	Other STIs	Chancroid	28	9.6%
		Herpes Simplex	14	4.8%
Know about contraceptive methods	Oral contraceptives	Yes	279	77.3%
		No	82	22.7%
	Injectibles	Yes	330	91.4%
		No	31	8.6%
	Implant	Yes	268	74.2%
		No	93	25.8%
	Other contraceptives	IUCD	52	14.4%
		Condom	13	3.6%
		Emergency contraceptives	4	1.1%
		Safe period method	2	0.6%

**Table 3:** distribution of attitude of parents towards RH discussion with adolescents in Debre Markos, 2019

Questions asked		Frequency	Percentage
Parents and their adolescents should discuss on RH	Agree	415	87.2%
	Disagree	32	6.7%
	Not sure	29	6.1%
Discussion about RH issues helps delay first sex	Agree	350	73.2%
	Disagree	37	7.7%
	Not sure	91	19.0%
Unmarried couples must use a condom if they want to have sex	Agree	318	66.5%
	Disagree	88	18.4%
	Not sure	72	15.1%
Condom prevents STIs and HIV/AIDS	Agree	350	73.2%
	Disagree	53	11.1%
	Not sure	75	15.7%
Contraceptives prevent unwanted pregnancy	Agree	342	71.7%
	Disagree	34	7.1%
	Not sure	101	21.2%

**Table 4:** discussion topics for parent-adolescent discussion in Debre Markos, March 2019

Topics of discussion	Categories	Frequency	Percentage
Discussion about RH issues is important	Yes	420	87.9%
	No	58	12.1%
Discuss contraceptive methods	Yes	176	36.8%
	No	302	63.2%
Discuss STIs and HIV/AIDS	Yes	243	50.8%
	No	235	49.2%
Discuss sexual intercourse	Yes	103	21.5%
	No	375	78.5%
Discuss unwanted pregnancy	Yes	184	38.5%
	No	294	61.5%
Discuss abstinence	Yes	217	45.4%
	No	261	54.6%
Discuss condom use	Yes	66	13.8%
	No	412	86.2%
Discuss changes during puberty	Yes	198	41.4%
	No	280	58.6%
Discuss menstrual cycles	Yes	173	36.2%
	No	305	63.8%
Ever discussed at least three components of RH	**Yes**	**264**	**55.2%**
	**No**	**214**	**44.8%**

**Factors associated with parent-adolescent discussion on RH Issues:** the bivariable analysis was employed to indicate the association between parent-adolescent discussion on RH issues and the independent variables individually. Sex, age, educational status, occupation, marital status, income, knowledge on RH issues, and attitude toward a discussion on RH issues were associated with parent-adolescent discussion on the bivariable analysis. These variables which had a p-value of less than 0.25 on the bivariable analysis were entered into the multivariable analysis. Age, sex, and income were significantly associated variables with parents' discussion with adolescents about RH issues in multivariable analysis. Parents aged 45-54 years were 2.37(AOR=2.37, 95% CI: 1.28-4.39) times, and parents aged 55-64 were 2.54(AOR=2.54, 95% CI: 1.15-5.56) times more likely to discuss RH issues than parents aged 25-34years. It was also shown that male parents were 49% (AOR= 0.51, 95% CI: 0.29-0.89) reduced to discuss RH issues with their adolescent children than female parents. Also, that parents who had a monthly income of above 158 USD were 3.308(AOR=3.308, 95% CI: 1.79-6.12) times more likely to discuss RH issues with their adolescent children than parents who had a monthly income of below 53.4 USD (Table 5).

**Table 5:** bivariate and multivariate analysis for barriers of parent-adolescent discussion among parents in Debre Markos, East Gojjam, Ethiopia, March 2019

Variables		Discussed		COR (95%)	AOR (95% CI)
		Yes	No		
**Age**	25-34	51	54	**1**	1
	35-44	101	111	**1.962(1.192-3.229)**	0.781(0.216-2.821)
	45-54	32	69	**2.036(1.154-3.592)**	**2.37( 1.28-4.39)**
	55-64	18	31	**1.252(1.612-2.563)**	**2.54(1.15-5.56)**
	Above 65	5	6	1.797(0.510-6.326)	0.486(0.110-2.901)
**Sex**	Female	149	208	**1**	**1**
	Male	58	63	**0. 22(0.114-0. 77)**	**0.51( 0.29-0.89)**
**Educational status**	Uneducated	47	78	0.657(0.363-01.192)	1.011(0.446-2.293)
	Read and write	36	45	0.873(0.458-1.662)	1.197(0.530-2.705)
	Primary school	32	44	0.793(0.412-1.529)	0.917(0.405-0.074)
	Secondary school	33	36	1	1
	Diploma	34	37	1.002(0.516-1.946)	1.178(0.525-2.647)
	Degree and above	25	31	0.880(0.434- 1.785)	1.094(0.513-2.336)
**Occupation**	Housewife	80	134	1	1
	Employed	50	61	1.308(0.823-2.077)	1.182(0.654-2.135)
	Merchant	53	56	1.510(0.949-2.402)	1.333(0.773-2.299)
	Other occupations	20	20	1.595(0.810-3.140)	1.603(0.748-3.439)
**Income**	<53.4 USD	50	68	**1**	**1**
	53.4-106.8 USD	87	78	1.517(0.942-2.442)	1.246(0.747-2.078)
	106.9-158 USD	30	49	0.833(0.465-1.491)	0.693(0.371-1.294)
	>158 USD	40	76	**1.67(1.122-2.150)**	**3.31( 1.79-6.12)**
**Knowledge of parents**	Not knowledgeable	93	154	**1**	**1**
	Knowledgeable	114	117	1.613(0.121-2.323)	1.574(0.017-2.459)
**Attitude of parents**	Negative attitude	63	121	1	1
	Positive attitude	144	150	0.144(0.260-1.698)	0.203(0.151-2.599)

## Discussion

In this study, it was found that parent-adolescent discussion on RH issues was 55.2% which was in line with studies conducted in Boditi (40.7%) [19] and Yirgalem (59.1%) [20] Southern Ethiopia. But It was higher than the results from the previously conducted research in Debre Markos, East Gojjam, Ethiopia (28.9%) [23], in Awabel, East Gojjam, Ethiopia (25.3%) [24], in Fiche, North Showa, Ethiopia (31.2%) [17], in Harar, Eastern Ethiopia (28.76%) [21], in Rwanda (29%) [25]. This discrepancy could be attributed to the differences in sample sizes in the studies and sociodemographic differences in the regions.

This study has revealed that parents aged 45-54 years were 2.37 times and parents aged 55-64 were 2.54 times more likely to discuss RH issues than parents aged 25-34years. This finding was different from the result found in a study in Rwanda, which stated [25].

It was also shown that male parents were 49% reduced to discuss RH issues with their adolescent children than female parents. This was similar to the result from the study in Rwanda; 53% of males did not discuss sexual matters compared to the females. This could be attributed to the fact that mothers are consistently noted as proactive in broaching sexuality talks, they cover more topics and they exhibit more comfort when discussing those topics [26].

It was also shown that parents who had a monthly income of above 158 USD were 3.31 times more likely to discuss RH issues with their adolescent children than parents who had a monthly income of less than 53.4 USD. This result was similar to the findings from the studies in Harar, Eastern Ethiopia [21], and Rwanda [25] which stated parents who had higher income were more likely to discuss RH issues than parents who had lower income. This could be due to the difficulties parents of lower-income find to have time to spend with their adolescent children due to the increased working hours so that they will be less close to their children which curtails the discussion [25, 27].

## Conclusion

This study has revealed that more than half of the parents did not discuss RH issues with their adolescent children. Age 45-54 and 55-64 years, male parents, and higher incomes were the factors that allowed parent-adolescent discussion on RH issues.

### What is known about this topic

- *Open positive parent-adolescent discussion on reproductive health issues is important to the adolescents, family, and society because it reduces risky sexual behaviours of the adolescents;*
- *Adolescents are susceptible to sexual risk-taking because of social pressures, mixed messages about sexuality, as well as having limited resources, or support to protect themselves from unsafe sex*.

### What this study adds

- *Male parents were 49% reduced to discuss RH issues with their adolescent children than female parents;*
- *Parents who had a monthly income of above 158 USD were 3.308 times more likely to discuss RH issues with their adolescent children than parents who had a monthly income of below 53.4 USD;*
- *Beyond half of the parents ever discussed at least three components of reproductive health issues with their adolescent children*.

## References

[1] Petersen AC, Hamburg BA (1986). Adolescence: A developmental approach to problems and psychopathology. Behavior Therapy.

[2] Brizio A, Gabbatore I, Tirassa M, Bosco FM (2015). “No more a child, not yet an adult”: studying social cognition in adolescence. Front Psychol.

[3] Ausubel D (1954). Theory and problems of adolescent development. iUniverse.

[4] Rosenthal DA, Hall C, Moore SM (1992). AIDS, adolescents, and sexual risk taking: A test of the Health Belief Model. Australian Psychologist.

[5] WHO (2012). Making health services adolescent friendly: Developing national quality standards for adolescent friendly health services. Department of Maternal, Newborn, Child and Adolescent Health.

[6] Kuzma EK, Peters RM (2016). Adolescent vulnerability, sexual health, and the NP´s role in Health Advocacy. Journal of American Association of Nurse Practitioners.

[7] Usonwu I, Ahmad R, Curtis-Tyler K (2021). Parent-adolescent communication on adolescent sexual and reproductive health in sub-Saharan Africa: a qualitative review and thematic synthesis. Reprod Health.

[8] Bastien S, Kajula LJ, Muhwezi WW (2011). A review of studies of parent-child communication about sexuality and HIV/AIDS in sub-Saharan Africa. Reprod Health.

[9] Kamangu AA, John MR, Nyakoki SJ (2016). Barriers to parent-child communication on sexual and reproductive health issues in East Africa: A review of qualitative research in four countries. Journal of African Studies and Development.

[10] Seif SA, Kohi TW (2014). Caretaker-Adolescent Communication on Sexuality and Reproductive Health: My Perceptions Matter; A Qualitative Study on Adolescents´ Perspectives in Ungunja, Zanzibar. Scientific Research Publishing.

[11] Tapert SF, Aarons GA, Sedlar GR, Brown SA (2001). Adolescent substance use and sexual risk-taking behavior. J Adolesc Health.

[12] Serovich JM, Greene K (1997). Predictors of adolescent sexual risk taking behaviors which put them at risk for contracting HIV. Journal of youth and adolescence.

[13] Jessica L, Morris HR (2015). Adolescent Sexual and Reproductive Health: The Global Challenges. International Journal of Gynecology and Obstetrics.

[14] Mengesha Kassie A, Beletew Abate B, Wudu Kassaw M, Gebremeskel Aragie T (2020). Prevalence of underweight and its associated factors among reproductive age group women in ethiopia: analysis of the 2016 Ethiopian demographic and health survey data. J Environ Public Health.

[15] CSAoE ICF (2017). Ethiopia Demographic and Health Survey, Addis Ababa, Ethiopia and Rockville, Merryland, USA: CSA and ICF.

[16] Collins T, Yusuf Y, Tesfahun H, Dejene S (2013). A Rights-Based Approach to Adolescent and Youth Development. Ethiopia, HLSP.

[17] Feyissa M (2018). Parent Adolescent Sexual and Reproductive Health Communication and Associated Factors among Secondary and Preparatory School Students in Fiche Town, North Shoa, Oromia Regional State, Ethiopia. HIA Africa Thesis Bank.

[18] Busi S, Chea N (2017). Barriers of Discussion Concerning Sexual and Reproductive Health Issues Among Adolescents and Parents, Hawassa, SNNPR, Ethiopia. Biomed J Sci & Tech Res.

[19] Fanta M, Lemma S, Sagaro GG, Meskele M (2016). Factors associated with adolescent-parent communication regarding reproductive health issues, among high school and preparatory students in Boditi town, Southern Ethiopia: a cross-sectional study. Patient Intelligence.

[20] Yohannes Z, Tsegaye B (2015). Barriers of Parent-Adolescent Communication on Sexual and Reproductive Health Issues among Secondary and Preparatory School Students in Yirgalem, Town, South Ethiopia. Fam Med Med Sci Res.

[21] Yadeta TA, Bedane HK, Tura AK (2014). Factors Affecting Parent-Adolescent Discussion on Reproductive Health Issues in Harar, Eastern Ethiopia: A Cross-Sectional Study. J Environ Public Health.

[22] (2018). 2011's Debre Markos City's Population Size by Five Years Interval. Debre Markos Ethiopia: Debre Markos City Administration.

[23] Mekuanint Taddele DJ, Alebachew Hunie (2017). Level of Parent Adolescent Communication on Sexual and Reproductive Health Issues and Associted Factors among Debre Markos Preparatory School Students, in Debre Markos, East Gojjam zone, Ethiopia. Universal Journal of Public Health.

[24] Ayehu A, Kassaw T, Hailu G (2016). Young people´s parental discussion about sexual and reproductive health issues and its associated factors in Awabel woreda, Northwest Ethiopia. Reprod Health.

[25] Bushaija E, Sunday F, Asingizwe D, Olayo R, Abong´o B (2013). Factors that Hinder Parents from the Communicating of Sexual Matters with Adolescents in Rwanda. Rwanda Journal of Health Sciences.

[26] Martin KA, Luke K (2010). Gender Differences in the ABC's of the Birds and the Bees: What Mothers Teach Yong Children About Sexuality and Reproduction. Sex Roles.

[27] Robinson M Barriers and Bridges to Communication.

